# Inhibitory Activity of Antibacterial Mouthwashes and Antiseptic Substances against Neisseria gonorrhoeae

**DOI:** 10.1128/aac.00042-22

**Published:** 2022-05-17

**Authors:** Eloise Williams, Bowen Zhang, Eric P. F. Chow, Socheata Chea, Tiffany R. Phillips, Kate Maddaford, Marcelina Krysiak, Yi Nong, Helen Stefanatos, Shivani Pasricha, Christopher K. Fairley, Deborah A. Wiliamson

**Affiliations:** a Department of Microbiology, Royal Melbourne Hospital, Melbourne, Victoria, Australia; b Department of Infectious Diseases, University of Melbourne at the Peter Doherty Institute, Melbourne, Victoria, Australia; c Melbourne Sexual Health Centre, Alfred Health, Melbourne, Victoria, Australia; d Central Clinical School, Monash University, Melbourne, Victoria, Australia; e Melbourne School of Population and Global Health, The University of Melbourne, Carlton, Victoria, Australia; f Department of Microbiology and Immunology, University of Melbourne at the Peter Doherty Institute, Melbourne, Victoria, Australia; g Victorian Infectious Diseases Reference Laboratory, Royal Melbourne Hospital at the Peter Doherty Institute, Melbourne, Victoria, Australia

**Keywords:** *Neisseria gonorrhoeae*, prevention, public health, sexually transmitted diseases

## Abstract

Improved treatment and prevention strategies, such as antimicrobial mouthwashes, may be important for addressing the public health threat of antimicrobial-resistant Neisseria gonorrhoeae. Here, we describe the activity of seven common antibacterial mouthwashes and antiseptics against N. gonorrhoeae isolates, incorporating the use of a human saliva test matrix. Our data demonstrate that antibacterial mouthwashes and antiseptics vary in their ability to inhibit the *in vitro* growth of N. gonorrhoeae and saliva may impact this inhibitory activity.

## INTRODUCTION

Antimicrobial resistance (AMR) in Neisseria gonorrhoeae is an urgent public health threat (1). The oropharynx is a critical site for gonorrhea transmission and the development of AMR (2, 3). Evidence also suggests that pharyngeal infections are more difficult to cure than urogenital infections (4). Novel strategies for the prevention and treatment of N. gonorrhoeae at this site are required, as traditional prevention strategies, such as condoms, are rarely used for oral sexual contact (5).

Some mouthwashes have *in vitro* bactericidal activity against N. gonorrhoeae (6, 7). However, two recent randomized controlled trials using mouthwash did not demonstrate a difference in the incidence of oropharyngeal gonorrhea in individuals receiving a daily preventative mouthwash over a 3-month period (8, 9). In addition, a recent study investigating the efficacy of a 14-day course of mouthwash twice daily compared with antibiotics for the treatment of oropharyngeal gonorrhea was stopped early due to a high failure rate in the mouthwash arm (10). These results demonstrate the need for further studies using mouthwash to further characterize the complex interplay of host and pathogen in the prevention and treatment of N. gonorrhoeae. Here, we assessed the *in vitro* inhibitory activity of seven common antibacterial mouthwashes and antiseptics against N. gonorrhoeae.

Six well-characterized isolates of N. gonorrhoeae were used, specifically World Health Organization (WHO) strain F, WHO strain K, WHO strain V, WHO strain X, WHO strain Y (11), and ATCC 49226 (12) (ATCC, Manassas, VA). These isolates were selected to ensure that a diverse range of N. gonorrhoeae AMR profiles were assessed (see Table S1 in the supplemental material). We tested commercial mouthwash products (Listerine Cool Mint, Listerine Zero, and Biotene Dry Mouth Relief) as well as 20% ethanol, 30% ethanol, 0.2% chlorhexidine, and 1% povodine iodine. Phosphate buffered saline (PBS) was included as a negative control (see Table S2 in the supplemental material). In addition, 40 mL of donor saliva from two healthy adult volunteers was pooled and tested negative for N. gonorrhoeae by nucleic acid amplification testing (Xpert CT/NG; Cepheid, Sunnyvale, CA). The saliva matrix was subsequently diluted in a 1:1 ratio with PBS, aliquoted into 400-μL samples, and stored at −80°C, such that each saliva matrix underwent a single freeze-thaw prior to use.

First, to establish that PBS was a suitable liquid medium for assessing the short-term growth of N. gonorrhoeae, we compared the viability of each N. gonorrhoeae strain in PBS and Giolitti-Cantoni (GC) broth (Media Preparation Unit, University of Melbourne, Parkville, Australia). Growth curves were performed in triplicate using a 10^5^ CFU/mL concentration of each N. gonorrhoeae isolate incubated in PBS and GC broth at ambient temperature for 24 h (see Supplementary Methods). For each N. gonorrhoeae strain, there was no significant difference in growth over 24 h in PBS compared with in GC broth (see Fig. S1 in the supplemental material).

Second, the growth of each N. gonorrhoeae strain in combination with each of the various antibacterial substances was assessed at different time points (Supplementary Methods). For each gonococcal isolate, we first assessed the growth of a 10^6^ CFU/mL concentration of each N. gonorrhoeae strain in PBS with the various antibacterial substances over a 30-min period. We then assessed the growth of a 10^6^ CFU/mL concentration of each N. gonorrhoeae strain in saliva with the antibacterial substances over a 30-min period and compared the results with growth in PBS. Although mouthwashes are typically used for less than 1 min, *in vitro* contact times of up to 30 min were included in this study due to the ability of a number of antibacterial mouthwashes and antiseptic substances to maintain antibacterial properties in the oral environment for up to 6 h after 10 to 30 s of use (13, 14). For each experiment, subcultures were taken from the liquid medium and plated onto GC + vancomycin, colistin, nystatin, and trimethoprim (VCNT) agar (Media Preparation Unit, University of Melbourne, Parkville, Australia) at 30 s, 60 s, 5 min, and 30 min (see Fig. S2 in the supplemental material). This process was performed in triplicate for each N. gonorrhoeae strain/antibacterial substance combination, with colony counts performed at each time point and after 48 h of incubation at 37°C in 5% CO_2_. Additional testing was conducted to confirm that the antibiotic-containing media did not impact testing results (Supplementary Methods).

Testing was performed by a single operator (B.Z.) and growth assessments recorded by two observers (B.Z. and E.W.). A comparison between saliva and PBS pairs was performed using a two-way ANOVA test, with a *P* value of <0.05 considered significant. Statistical analysis and data visualization were performed using GraphPad Prism version 9.1.2.

**FIG 1:**
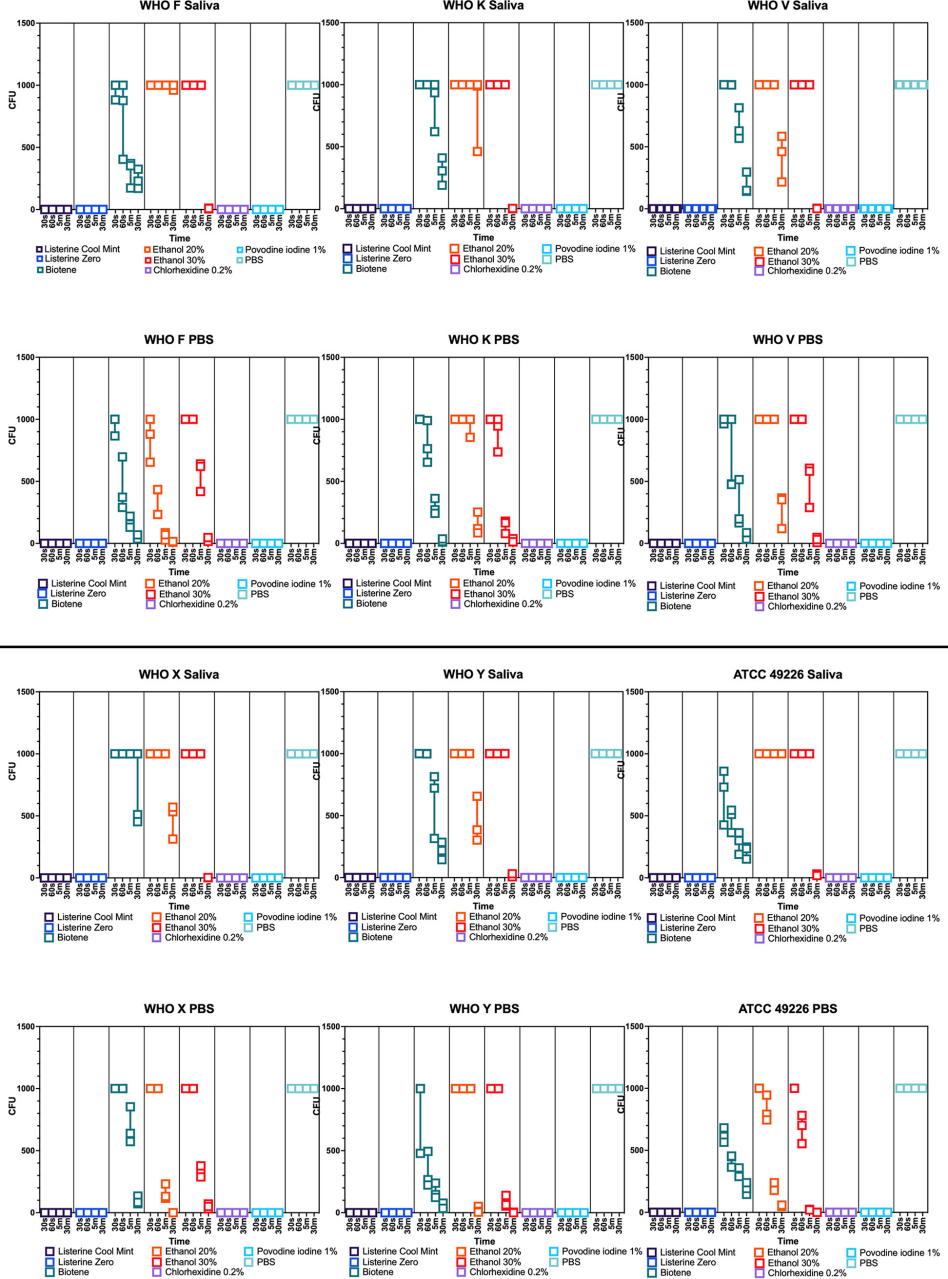
Time-kill kinetics of antibacterial mouthwashes and antiseptic substances against Neisseria gonorrhoeae isolates. Scatterplot demonstrating the CFU of three replicates (each box represents a single replicate) per N. gonorrhoeae isolate and antibacterial substance combination over time.

**FIG 2:**
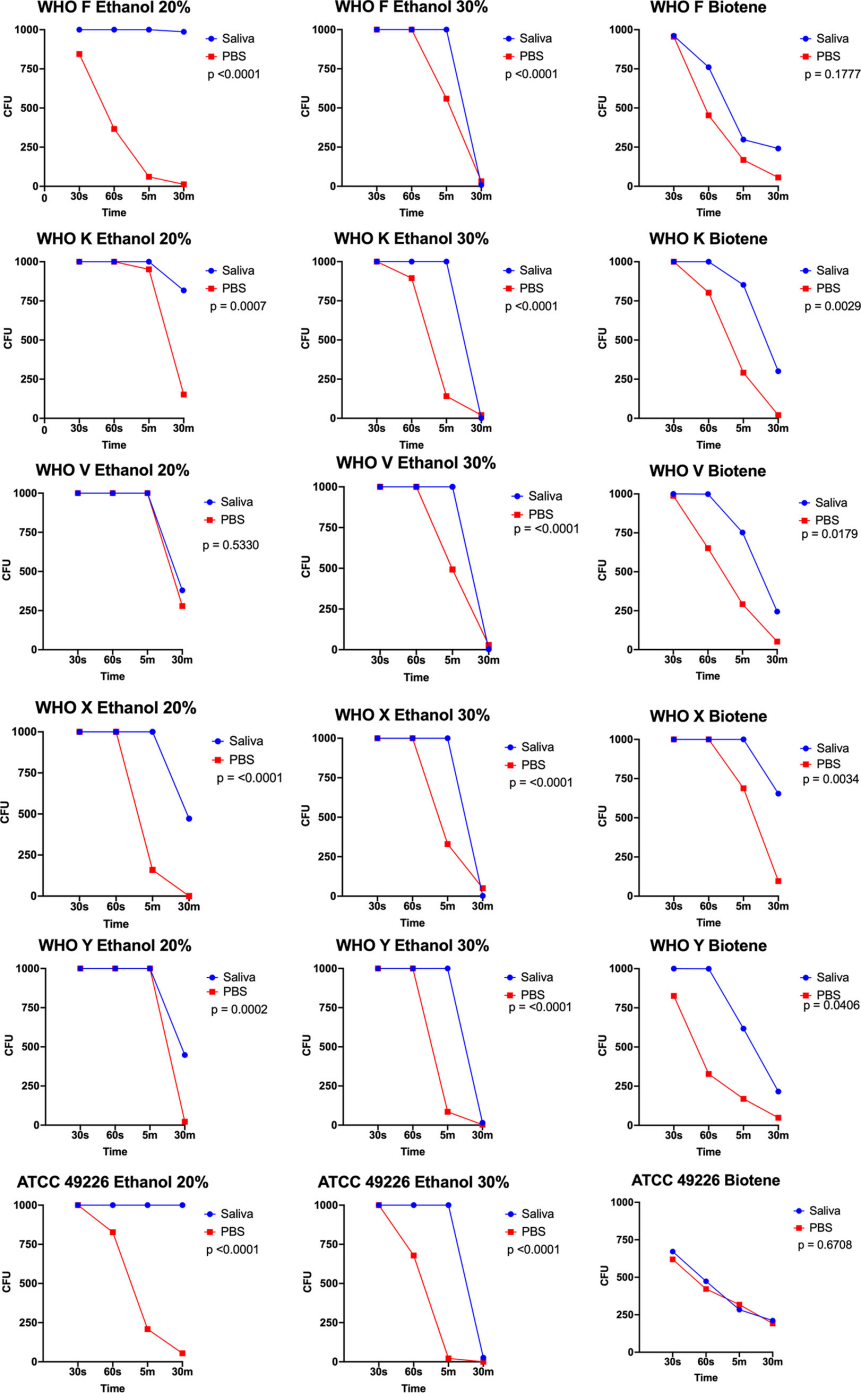
Neisseria gonorrhoeae inhibition with ethanol and Biotene in combination with saliva or phosphate-buffered saline over time. Line graph demonstrates the mean CFU of the three replicates per N. gonorrhoeae isolate and antibacterial substance combination over time.

When combined with PBS and Listerine Cool Mint, Listerine Zero, 0.2% chlorhexidine, or 1% povodine iodine, N. gonorrhoeae was fully inhibited (defined as no visible growth) at all time points. In contrast, when combined with PBS and Biotene Dry Mouth Relief, 20% ethanol, or 30% ethanol, N. gonorrhoeae remained viable, although increased inhibition was observed with increased contact time (Fig. 1). Similar results were obtained when N. gonorrhoeae was combined with saliva and Listerine Cool Mint, Listerine Zero, 0.2% chlorhexidine, 1% povodine iodine, or Biotene Dry Mouth Relief (Fig. 1), and there was no significant difference between results obtained in saliva and PBS (Fig. 2). In contrast, N. gonorrhoeae was significantly less inhibited when exposed to ethanol at 20% or 30% in saliva compared with that in PBS for all isolate/ethanol combinations except N. gonorrhoeae WHO V and 20% ethanol (Fig. 2).

Compared with a previous study where saliva demonstrated inhibitory activity against N. gonorrhoeae, likely mediated by salivary alpha-amylase (15), in our study, saliva itself did not demonstrate inhibitory activity against N. gonorrhoeae. An additional study suggested the inhibitory activity of amylase described in the initial work was artefactual due to the hydrolysis of starch within the GC media rather than inhibitory activity against N. gonorrhoeae, with the findings not reproducible on solid chocolate agar or in PBS (16).

The reasons for the reduced inhibition of N. gonorrhoeae in the salivary and ethanol combinations compared with PBS and ethanol are unclear, but a previous study has noted a high relative abundance of *Neisseria* spp. in the oral microbiome of subjects with higher alcohol consumption (17). In addition, a study of the antibacterial activity of essential oils on oral biofilm showed that alcohol-free essential oil formulations were better at reducing biofilm thickness and covering grade than those with alcohol (14). There are over 45 antimicrobial peptides in human saliva, including cationic peptides, peroxidases, and lysozymes; inhibition of these peptides by ethanol may play a role in promoting N. gonorrhoeae growth (18). Further work is required to investigate the impact of ethanol on normal salivary constituents and the subsequent impact on the oral microbiome. Importantly, the one commercially available alcohol-containing mouthwash included in this study (Listerine Cool Mint with 22% alcohol) demonstrated complete inhibition of N. gonorrhoeae in both the PBS and saliva matrices at all time points, likely due to the inhibitory activity of the other antibacterial constituents in the preparation. These findings are consistent with earlier studies (6, 7). Further studies would benefit from the inclusion of additional commercially available alcohol-containing mouthwashes to assess the impact of saliva on the inhibition of N. gonorrhoeae in these preparations.

In summary, our data demonstrate that antibacterial mouthwashes and antiseptic substances vary in their ability to inhibit the *in vitro* growth of N. gonorrhoeae. This variability is an important consideration when assessing antibacterial mouthwashes for use in future N. gonorrhoeae treatment or prevention clinical studies. To date, there have been no studies incorporating human saliva into *in vitro* studies assessing the antigonococcal activity of antibacterial mouthwashes and oral antiseptic substances. This work demonstrates that saliva may have an impact on the inhibitory activity of some antibacterial substances. We therefore suggest that future *in vitro* studies investigating the activity of antibacterial mouthwashes and oral antiseptic substances on N. gonorrhoeae incorporate saliva into the test matrix.
