# Supplementary material for: Inhibitory Activity of Antibacterial Mouthwashes and Antiseptic Substances against Neisseria gonorrhoeae
Source: Antimicrob Agents Chemother. 2022 May 17;66(6):e00042-22. doi: 10.1128/aac.00042-22 (PMC9211398; doi:10.1128/aac.00042-22)
Supplement: Supplemental file 1 — Supplemental methods, Fig. S1 to S3, and Tables S1 and S2. Download aac.00042-22-s0001.pdf, PDF file, 0.7 MB [file aac.00042-22-s0001.pdf]

SUPPLEMENTARY MATERIAL

Supplementary Methods

N. gonorrhoeae growth curves

In order to establish that PBS at ambient temperature was a suitable liquid medium for assessing short-term growth of *N. gonorrhoeae*, growth curves were performed in conditions that replicated those used for the *in vitro* assessment of *N. gonorrhoeae* growth performed in this study. Growth curves were performed by bringing into suspension each gonococcal isolate in 2ml PBS at 0.45-0.55 McFarland turbidity, corresponding to a concentration of 10^8 CFU/ml. From the first suspension, 10ul was added to 990ul PBS; 100ul from this second suspension to 900ul PBS, resulting in a third suspension with a concentration of 10^5 CFU/ml. At timepoints 0, 30 minutes, 1 hour, 2 hours, 4 hours, 6 hours and 24 hours at ambient temperature (20 +/- 5°C), 10ul aliquots from the third suspension were subcultured onto GC+VCNT agar (GC agar with vancomycin, colistin, nystatin and trimethoprim; Media Preparation Unit, University of Melbourne, Parkville, Australia) and incubated for 48 hours in a 5 +/- 1% CO2 environment at 35 +/- 2°C. In addition, 10ul from the first suspension was added to 990ul GC broth; and 100ul from this second suspension was added to 900ul GC broth as a growth control. This sample also had 10ul aliquots subcultured to GC+VCNT agar at timepoints 0, 30 minutes, 1 hour, 2 hours, 4 hours, 6 hours and 24 hours in the same conditions as above. This process was repeated three times for each *N. gonorrhoeae* isolate, producing results in triplicate for each of the three *N. gonorrhoeae* isolates combined with PBS and GC broth, respectively. Ambient temperature was selected for the initial incubation as each gonococcal isolate was combined with antibacterial mouthwash or antiseptic substances at ambient temperature for up to 30 minutes in the *in vitro* study described below, followed by subculture to solid media and incubation at

35 +/- 2°C. Comparison between growth in GC broth and PBS for each isolate was performed using a two-way ANOVA test, with a p value of <0.05 considered significant. Statistical analysis was performed using GraphPad Prism Version 9.1.2.

Assessment of N. gonorrhoeae growth in combination with antibacterial mouthwashes over time

Each gonococcal isolate was suspended in 2ml PBS at 0.45-0.55 McFarland turbidity, corresponding to a concentration of 10^8 CFU/ml. From the first suspension, 100ul was then added to 900ul PBS, corresponding to a concentration of 10^7 CFU/ml. From the second suspension, 100ul was then added to 400ul of pooled saliva matrix and 500ul of the antibacterial substance being tested and mixed for 10 seconds, resulting in a final concentration of 10^6 CFU/ml. After 30 seconds, 60 seconds, 5 minutes and 30 minutes at ambient temperature (20 +/- 5C), 10ul aliquots were subcultured onto GC+VCNT agar (GC agar with vancomycin, colistin, nystatin and trimethoprim; Media Preparation Unit, University of Melbourne, Parkville, Australia) and incubated for 48 hours in a 5% CO2 at 37°C. After the 60-second subculture was complete, a further 100ul aliquot from the second suspension was added to 400ul PBS and 500ul of the substance being tested and pipette-mixed for 10 seconds. This sample also had 10ul aliquots subcultured onto GC+VCNT agar after 30 seconds, 60 seconds, 5 minutes and 30 minutes and incubated in the same conditions as above. Selective gonococcal media (GC+VCNT agar) was used in this work in order to selectively inhibit the normal oral flora and allow accurate assessment of colony forming units.

Assessment of N. gonorrhoeae growth on antibiotic-containing media compared to media without antibiotics

A standard gonococcal selective media (GC+VCNT agar; Media Preparation Unit, University of Melbourne, Parkville, Australia) containing four different antibiotics (vancomycin, colistin, nystatin and trimethoprim) was used for assessment of *N. gonorrhoeae* viability in combination with various antibacterial mouthwashes and antiseptic substances in this study. This method was chosen in order to selectively inhibit the growth of normal oral flora to allow accurate assessment of *N. gonorrhoeae* colony forming units. To establish whether the antibiotic-containing GC media used in the assessment of *N. gonorrhoeae* growth in combination with various antibacterial substances impacted the growth of *N. gonorrhoeae* in this study, we compared the growth of *N. gonorrhoeae* strains WHO X and WHO Y on antibiotic-containing GC media compared to antibiotic-free GC media. Each gonococcal isolate was suspended in 2ml PBS at 0.45-0.55 McFarland turbidity, corresponding to a concentration of 10^8 CFU/ml. From the first suspension, 100ul was then added to 900ul PBS, corresponding to a concentration of 10^7 CFU/ml. From the second suspension, 100ul was then added to 400ul of PBS and 500ul of the antibacterial substance being tested (Listerine Cool Mint®, Listerine Zero® and Biotene Dry Mouth Relief®, 20% ethanol, 30% ethanol, 0.2% chlorhexidine, 1% povodine iodine and PBS as a negative control). After 30 seconds, 60 seconds, 5 minutes and 30 minutes at ambient temperature (20 +/- 5C), 10ul aliquots were subcultured onto GC+VCNT agar (GC agar with vancomycin, colistin, nystatin and trimethoprim; Media Preparation Unit, University of Melbourne, Parkville, Australia) and GC agar (Media Preparation Unit, University of Melbourne, Parkville, Australia), respectively and incubated for 48 hours in 5% CO2 at 37°C. This process was performed in triplicate for each *N. gonorrhoeae* strain / media type, with colony counts read for each time point after 48 hours of incubation.

Testing and growth assessments were performed by a single operator (MK). Comparison between saliva and PBS pairs was performed using a two-way ANOVA test, with a p value of

<0.05 considered significant. Statistical analysis and data visualisation was performed using GraphPad Prism Version 9.1.2.

Growth was completely inhibited at all timepoints on both GC+VCNT media and GC media without antibiotics for *N. gonorrhoeae* WHO strains X and Y combined with Listerine Cool Mint®, Listerine Zero®, povodine iodine 1% and chlorhexidine 0.2%. There was no significant difference in growth of *N. gonorrhoeae* WHO strains X and Y combined with ethanol 20%, ethanol 30% or PBS over the 30-minute period on GC agar containing antibiotics (vancomycin, colistin, nystatin and trimethoprim) compared to GC agar without antibiotics (Supplementary Figure 3). *N. gonorrhoeae* WHO strain X combined with Biotene Dry Mouth Relief® was significantly more inhibited when subcultured to GC+VCNT media compared to GC media without antibiotics, however there was no significant difference in inhibition for *N. gonorrhoeae* WHO strain Y combined with Biotene Dry Mouth Relief® (Supplementary Figure 3).

Supplementary Figure 1. Growth curves of *Neisseria gonorrhoeae* isolates in phosphate buffered saline and GC broth.

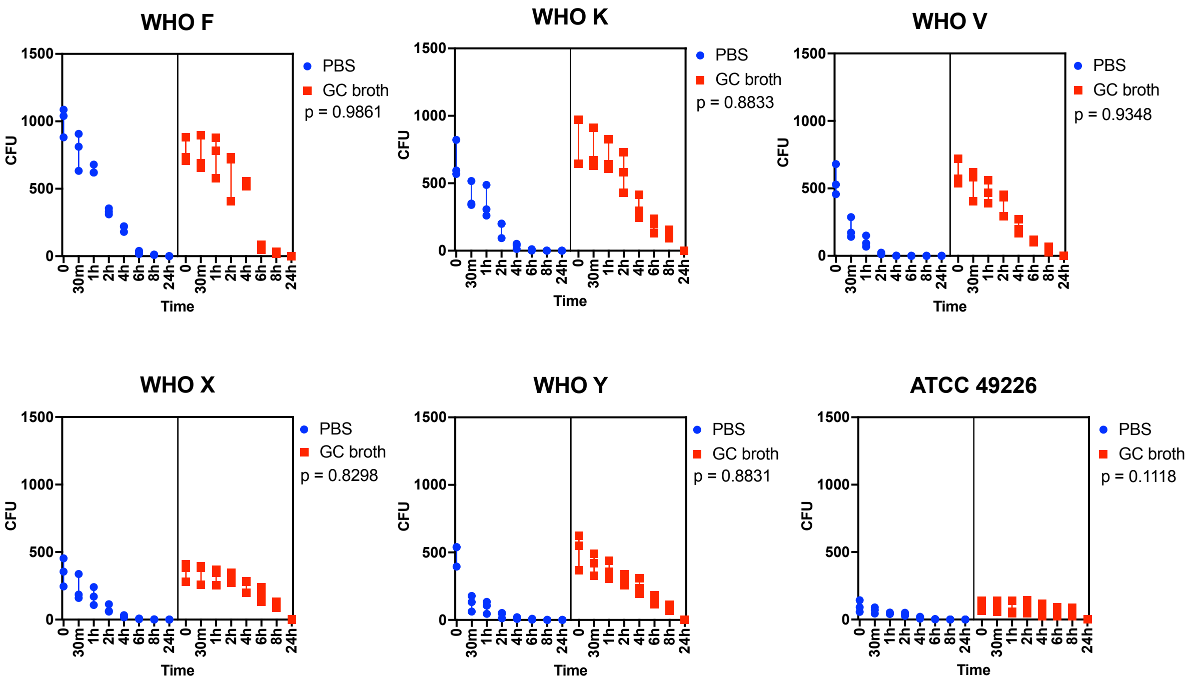

Scatter plot demonstrating results of three replicates (each symbol representing a single replicate) per *N. gonorrhoeae* isolate combined with either PBS or GC broth over time. CFU: colony forming units; m: minutes; s: seconds; PBS: phosphate buffered saline

Supplementary Figure 2. Schematic diagram demonstrating the method used to determine growth of *Neisseria gonorrhoeae* when exposed to each antibacterial substance in either PBS or saline.

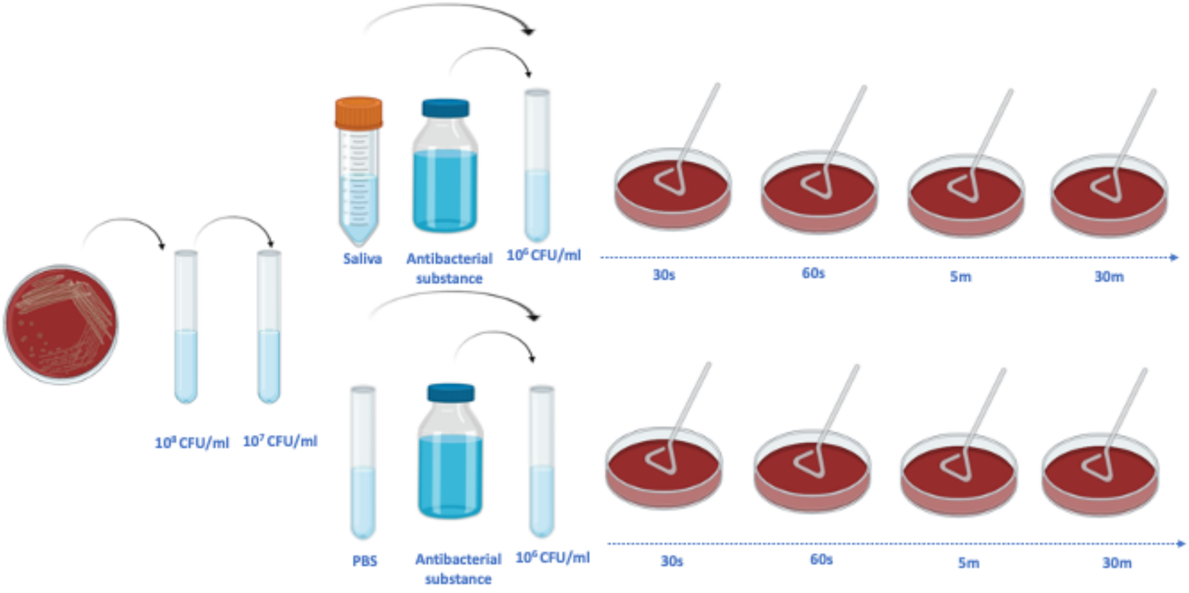

Supplementary Figure 3. ***N. gonorrhoeae*** **growth on antibiotic-containing GC media compared to GC media without antibiotics**

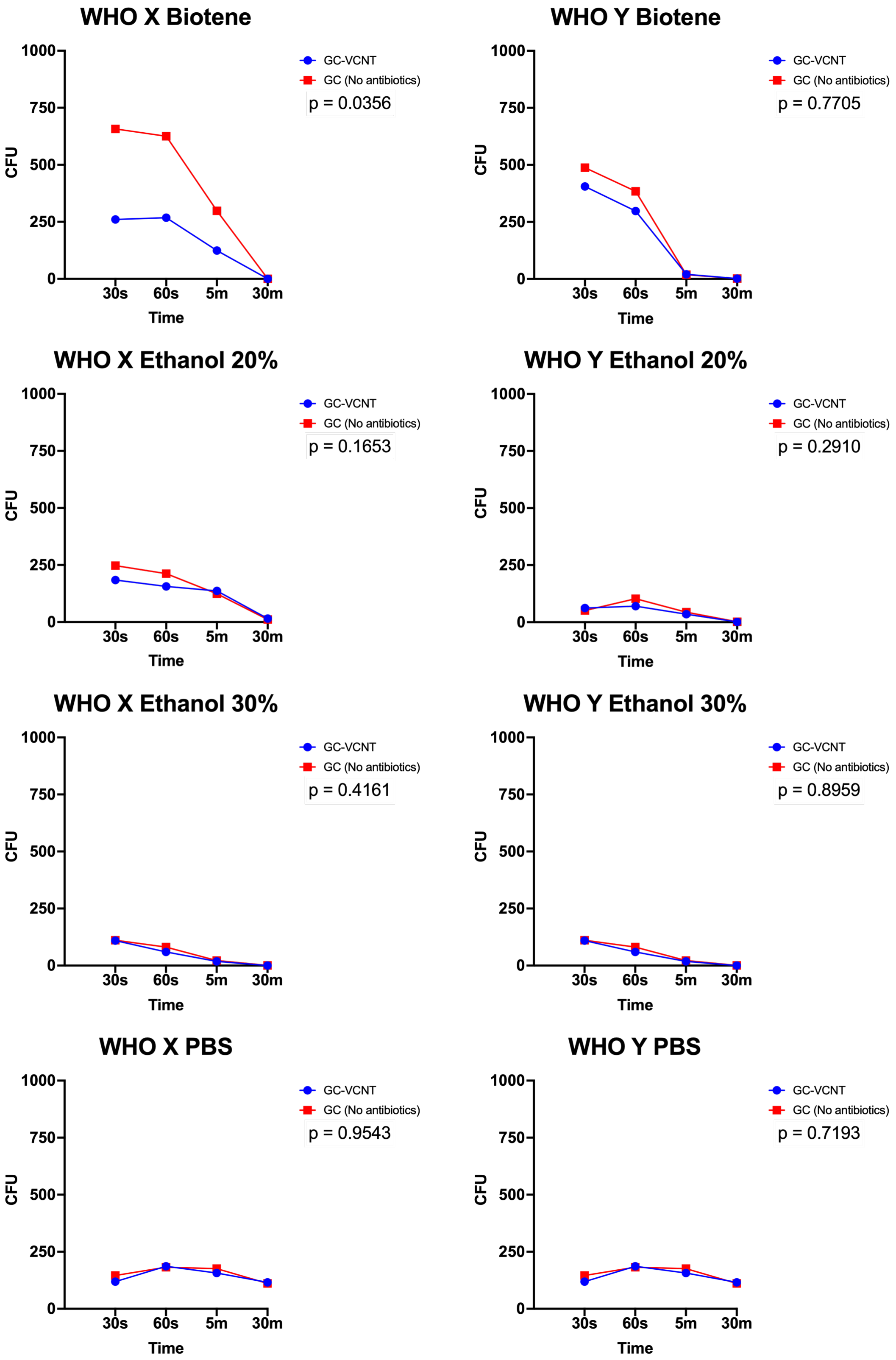

Supplementary Table 1. Antimicrobial susceptibility phenotype of *Neisseria gonorrhoeae* strains

**Antibiotics**	**WHO F**		**WHO K**		**WHO V**		**WHO X**		**WHO Y**		**ATCC 49226**	
**Result**	**I**	**MIC**	**I**	**MIC**	**I**	**MIC**	**I**	**MIC**	**I**	**MIC**	**I**	**MIC**
**Penicillin G**	S	0.032	R	2	R	>32	R	4	I	1	I	0.5
**Ceftriaxone**	S	<0.002	S	0.064	S	0.064	R	2	R	1	S	0.008
**Ertapenem**	-	0.004	-	0.125	-	0.004	-	0.064	-	0.064	-	0.012
**Azithromycin**	S	0.125	S	0.25	R	>256	I	0.5	R	1	S	0.5
**Tetracycline**	S	0.25	R	2	R	4	R	2	R	4	I	0.5
**Ciprofloxacin**	S	0.004	R	>32	R	>32	R	>32	R	>32	S	0.004
**Spectinomycin**	S	16	S	16	S	16	S	16	S	16	S	16
**Gentamicin**	-	4	-	4	-	8	-	4	-	8	-	4
**Solithromycin**	-	0.064	-	0.064	-	32	-	0.064	-	0.125	-	0.5
**Zoliflodacin**	-	0.032	-	0.064	-	0.064	-	0.064	-	0.064	-	0.25

Antimicrobial susceptibility phenotype of reference strains based on literature review[3,4,5].

Antimicrobial susceptibility phenotype of reference strains using CLSI agar dilution method[1].

ATCC: American Type Culture Collection; I: interpretation; MIC: minimum inhibitory concentration; WHO: World Health Organization.

Supplementary Table 2. Antibacterial mouthwashes and antiseptic substances used in this study

Substance	Alcohol	Ingredients
Biotène® Dry Mouth Relief	0%	Water, glycerin, xylitol, sorbitol, propylene glycol, poloxamer 407, sodium benzoate, hydroxyethylcellulose, methylparaben, propylparaben, flavour, sodium phosphate, disodium phosphate (GlaxoSmithKline, Brentford, UK)
Listerine® Zero	0%	Water, sorbitol, propylene glycol, sodium lauryl sulfate, poloxamer 407, eucalyptol, benzoic acid, sodium benzoate, methyl salicylate, thymol, sodium saccharin, menthol, sodium fluoride, flavour, sucralose, CI 42053 (Johnson and Johnson Pacific, Ultimo, Australia).
Listerine® Cool Mint	22%	Water, alcohol, sorbitol, poloxamer 407, benzoic acid, sodium saccharin, eucalyptol, flavour, thymol, methyl salicylate, sodium benzoate, menthol, CI 42053 (green 3) (Johnson and Johnson Pacific, Ultimo, Australia).
20% Ethanol	20%	20% ethanol, prepared by adding 2000ul of pure ethyl alcohol (Sigma-Aldorich®, Sheboygan Falls, USA) and 8000ul of distilled water
30% Ethanol	30%	30% ethanol, prepared by adding 3000ul of pure ethyl alcohol (Sigma-Aldorich®, Sheboygan Falls, USA) and 7000ul of distilled water
0.2% Chlorhexidine	0%	0.2% chlorhexidine, prepared by adding 50ul of chlorhexidine digluconate solution 20% in H_2O (Sigma-Aldorich®, Sheboygan Falls, USA) and 4950ul of distilled water

1% Povodine iodine	0%	1% povodine iodine, prepared by adding 1000ul of 10% povidone-iodine (Betadine®, Leusden, Netherlands) and 9000ul of distilled water
Phosphate buffered saline	0%	Saline, phosphate

References

1. Kim WJ, Higashi D, Goytia M, Rendon MA, Pilligua-Lucas M, Bronnimann M, McLean JA, Duncan J, Trees D, Jerse AE, So M. 2019. Commensal Neisseria kill Neisseria gonorrhoeae through a DNA-dependent mechanism. Cell Host Microbe 26:228-239 e8.

2. Aho EL, Ogle JM, Finck AM. 2020. The human microbiome as a focus of antibiotic Discovery: Neisseria mucosa Displays Activity Against Neisseria gonorrhoeae. Front Microbiol 11:577762.

3. Unemo M, Golparian D, Sanchez-Buso L, Grad Y, Jacobsson S, Ohnishi M, Lahra MM, Limnios A, Sikora AE, Wi T, Harris SR. 2016. The novel 2016 WHO *Neisseria gonorrhoeae* reference strains for global quality assurance of laboratory investigations: phenotypic, genetic and reference genome characterization. J Antimicrob Chemother 71:3096-3108.

4. Bharat A, Martin I, Zhanel GG, Mulvey MR. 2016. In vitro potency and combination testing of antimicrobial agents against *Neisseria gonorrhoeae.* J Infect Chemother 22:194-7.

5. Clinical and Laboratory Standards Institute (CLSI). Performance standards for antimicrobial susceptibility testing. 31st ed. CLSI supplement. Clinical and Laboratory Standards Institute, Wayne, Pennsylvania, USA, 2021.
